# Pathways to policy integration: a subsystem approach

**DOI:** 10.1007/s11077-022-09483-1

**Published:** 2022-11-26

**Authors:** Guillermo M. Cejudo, Philipp Trein

**Affiliations:** 1 Institute d’études Politiques, Université de Lausanne, Géopolis 4126, 1015 Lausanne, Switzerland; 2 Public Administration Department, Center for Research and Teaching in Economics (CIDE), Carretera México-Toluca 3655 Col. Lomas de Santa Fe, 01210, Mexico City, Mexico

**Keywords:** Policy coordination, Agenda-setting, Actor coalitions, Veto points, Policy implementation, Policy evaluation

## Abstract

Researchers in public policy and public administration agree that policy integration is a process. Nevertheless, scholars have given limited attention to political aspects that facilitate or impede integration. This paper aims at filling that gap, by looking at how different theories of the policy process can help in explaining the process of policy integration as shaped by policy subsystems. By building on insights from theories of the policy process, we develop pathways regarding adoption and implementation in policy integration that account for the politicization and the role of actors and subsystems in the policy process. Our main argument is that policy integration is in permanent political tension with the sectoral logic of policymaking, which predominantly happens between actors in subsystems. Policy integration is, thus, not a single moment when those tensions are solved once and for all, but a political process that requires deliberate efforts to overcome the pull toward sector-specific problem definition, policymaking, implementation, and evaluation.

## Introduction

Policy integration is a political process that entails the coordination of actors and agencies across policy subsystems, the combination of instruments from different policy sectors, as well as arrangements for their consistent implementation and evaluation, as a response to a complex policy problem that not one policy sector, policy instrument, or agency can solve (Candel & Biesbroek, 2016; Cejudo & Michel, 2017; Trein et al., 2021a, 2021b). This understanding has stimulated a growing strand of research in the policy sciences (Tosun & Lang, 2017; Trein et al., 2019), which has (re)discovered the importance of questions dealing with new and transversal policy goals when facing complex challenges, such as environmental protection, climate change, public health, gender equality, and migration, from established policy subsystems (Adelle & Russel, 2013; Daly, 2005; Ingold & Tosun, 2020; Jordan & Lenschow, 2010; Metz et al., 2020; van Breugel & Scholten, 2017; Varone et al., 2013).

Researchers have emphasized that policy integration must be understood as a process. Yet, the policy integration literature has essentially taken a problem-oriented design perspective on policymaking (Briassoulis, 2017; Howlett & del Rio, 2015) and has given limited attention to the political dynamics of policy integration. Indeed, a top-down, designed-oriented perspective has dominated the field. This bias has come at the expense of a more political perspective of policy integration.

In this paper, we propose that policy integration can benefit from studying the classical questions of policy analysis and the theories developed in the study of the policy process. Harnessing the insights from these theoretical approaches allows us to better explain the stability and change in the process of policy integration, notably how policies result from interactions among actors with interests, values, and preferences, and how, once enacted, policies are reshaped in the implementation process. Specifically, we contribute to this endeavor by using a policy subsystem perspective (Jochim & May, 2010; May et al., 2011) to analyze political obstacles to policy integration and examine pathways through which they may be overcome.

Policy subsystems (i.e., policy sectors or policy fields—we use the terms interchangeably) denote the presence of a community of diverse actors that are specialized in a particular policy problem, such as unemployment, economic growth, or environmental protection. These actors interact with other policy specialists about formulating and implementing policies related to the subsystem (Hill & Varone, 2021; Howlett et al., 2020; Knill & Tosun, 2020; Lasswell, 1970; Sabatier & Jenkins-Smith, 1993; Weible & Sabatier, 2018). Coordination between different policy sectors is often complicated due to conflicts between organizations, different sectoral policy styles (Cairney, 2021; Peters, 2015), and the relative autonomy of policy subsystems (Cairney & Weible, 2017).

We hold that policy integration is in permanent political tension with the sectoral (subsystem) logic of policymaking. These political dynamics create a pull toward fragmented policymaking, which affects the whole policy process, from identifying policy problems, building solutions, putting them in motion, and evaluating them. Policy integration is, thus, not a single moment when those tensions are solved once and for all, but a process that requires deliberate political efforts to overcome the pull toward a sector-specific policy process.

Rather than developing a new policy process theory tailored to the analysis of policy integration, we explore how existing theoretical frameworks about the policy process can explain why and how policy integration occurs. In using insights from specific policy process theories (Weible & Sabatier, 2018) that inform the policy subsystems’ perspective, we develop theoretical propositions regarding agenda-setting and decision-making as well as the implementation and evaluation of policy integration, which we embed into different pathways to achieve policy integration. The added value of this paper compared to other publications, which focus on policy integration as a process (e.g., Candel & Biesbroek, 2016; Cejudo & Michel, 2017), is that *we shed light on the politicization and the role of actors in the process of policy integration*. By doing so, we pave the way to develop an empirical research agenda on the political dynamics of policy integration.

This article provides a roadmap for studying the politics of policy integration. The next section defines policy integration and situates it in the current state of the literature. The politicization of policy integration section explains the crucial role of politicization in shaping the pathways toward policy integration, whereas the Outputs of the process: strategies and capacities for policy integration section details the possible outputs of the process. In the Agenda-setting and decision-making section, we address the question of how policy integration emerges as a solution to policy problems and develops propositions regarding agenda-setting and decision-making related to policy integration. In the Implementation and evaluation section, the paper uses policy theories to explain the challenges of implementing and evaluating policy integration across policy subsystems. In the conclusion, we return to the literature to discuss our propositions and offer some avenues for further research.

## Defining policy integration

Building on the recent literature, we define policy integration as a political process that entails actors and agencies coordinating across different policy subsystems, the coherent combination of instruments from different policy sectors, as well as arrangements for their consistent implementation and evaluation to address different dimensions of a complex problem (Candel & Biesbroek, 2016; Cejudo & Michel, 2017; Howlett & del Rio, 2015; Tosun & Lang, 2017; Trein & Maggetti, 2020; Trein et al., 2021a, 2021b). This definition excludes mere administrative coordination, intergovernmental cooperation, or collaboration across agencies where policy instruments and practices remain unchanged and involvement is discretionary and can be rescinded and negotiated (Agranoff, 2018). It is also different from policy accumulation, consisting of adding (layering) new instruments and targets to an existing policy portfolio, without redesigning the mix of instruments (Adam et al., 2018). And it is different from broad policy goal alignment: the mere acknowledgment that individual policies do not serve only their own purposes, but also address broader national, or even global goals (such as the United Nations Sustainable Development Goals), but without modifying the sector-specific logic of design and implementation. It is, first and foremost, a political process, where actors—whether politicians, interest groups, or bureaucrats—ideas and interests interact and compete, cooperate, and resist.

The process of policy integration, as explained by Candel & Biesbroek, (2016), is multi-layered and asynchronous. Understanding this process requires not a single overarching theory of policy integration, but the theory-informed analysis of specific sequences of events that shape how it unfolds. Nevertheless, up to now policy integration literature only implicitly (if at all) uses the logic of the policy process.^1^ Instead, scholars have focused on how instruments from multiple policy sectors are incorporated into an integrated policy, taking the perspective of policy design (Howlett & Mukherjee, 2018) or policy formulation (Howlett & Mukherjee, 2017). From a design perspective, it has been argued that policy integration requires that all instruments appropriately interact with each other, enabling them to potentially achieve such a goal (Briassoulis, 2017; Howlett & del Rio, 2015). Moreover, the coherence and congruence of the instruments that make up the policy mix must be preserved during implementation (Bolognesi & Pflieger, 2019; Bolognesi et al., 2021; Cejudo & Michel, 2021; Maor & Howlett, 2021).

Yet, policy integration is more than policy design and decision-making. Indeed, policy integration is a type of policy change by which policymakers link subsystems from different policy areas that follow their own logic (Tosun & Lang, 2017; Trein et al., 2019). Policy integration is a special form of policy change because it requires actors to continuously link policy subsystems at numerous points along the policy process. For example, the government may announce an integrated policy (agenda-setting) only to have it fragmented at the design stage or later when it is put into action. As with any other policy change, understanding how efforts to integrate policies are in tension with the sectoral logic requires explaining both why this logic usually prevails and how it is overcome by deliberate political interventions.

## The politicization of policy integration

In this paper, we unpack the process of policy integration and examine how actors within policy subsystems impact integrated policymaking during the policy process. Based on insights from theories of the policy process, we present different pathways to policy integration, showcasing the role of actors within policy subsystems in shaping the process of integration. Our premise is that, by definition, existing actors operating according to their own policy subsystems logic impact policymaking and undermine the efforts toward policy integration at every moment of the policy process: Actor networks within policy sectors impede the integration of new goals into different policy subsystems, a coherent integration policy instruments from different sectors, and the coordinated implementation and evaluation of policies from different subsystems.

The politicization of policy problems happens within and among policy subsystems. The maturation of public policies comes along with lock-in effects by which established actor networks hold power over agenda-setting and decision-making in policy subsystems (Baumgartner et al., 2018; Jenkins-Smith et al., 2018; Jones et al., 1993; McGee & Jones, 2019). Thus, we hold that policy integration is in constant tension with the sectoral structure and logic of policymaking, supported and defended by actors within subsystems (Hill & Varone, 2021; Howlett et al., 2020; Knill & Tosun, 2020; Peters, 2015; Weible & Sabatier, 2018). Following existing theories of the policy process (Weible & Sabatier, 2018), we take policy agendas as being sector-specific, policy instruments and goals defined within policy subsystem coalitions, and implementation and evaluation carried out by administrative agencies focused on their own priorities and targets. Therefore, the subsystem logic implies that any policy integration initiative may fall victim to the dominance of subsystem logic of policymaking, as actors are likely to dismantle, dilute, or neutralize efforts toward integrated policies during agenda-setting, decision-making, and implementation.

Nevertheless, the literature on policy integration has demonstrated that this sectoral approach might be overcome, and previous research has indicated how this occurs (Biesbroek & Candel, 2020; Cejudo & Michel, 2017; Dang et al., 2021; Trein et al., 2021a, 2021b; Vogeler et al., 2021). In this article, we generalize insights from this literature and identify political pathways to (and away from) policy integration, focusing on how policy subsystems shape the processes of *(1) agenda-setting and decision-making; and (2) implementation and evaluation* (Hill & Varone, 2021; Howlett et al., 2020; Knill & Tosun, 2020).

Regarding policy integration in the phase of agenda-setting and decision-making, we theorize the impact of the politicization of policy issues, in the policy process (Baumgartner et al., 2018; Culpepper, 2010; Feindt et al., 2020). A definition from the EU literature posits that politicization entails public attention to an issue (salience), “a polarization of opinion” and an “expansion of actors and audiences” dealing with the topic (de Wilde et al., 2016, p. 5). This view on politicization could apply to policy proposals integrating different subsystems or to more sector-specific policy proposals. Politicization is the opposite of depoliticization, which refers to attempts by “the state” to prevent important policy decisions from being discussed and questioned in the public arena (Wolf & Van Dooren, 2018; Wood, 2016, pp. 523–525) as well as to the broader reduction of political deliberation and conflict in the public and the private spheres (Fawcett et al., 2017, p. 12).

To theorize how actors politicize policy integration in agenda-setting and decision-making, we use the reference manual *Theories of the Policy Process*, which is an authoritative guide to the political analysis of the policy process (Weible & Sabatier, 2018). We focus on theories that clearly take a subsystems approach and can make a substantive explanatory contribution to understanding the politicization of policy integration: the Punctuated Equilibrium Framework, the Multiple Streams Framework, the Advocacy Coalition Framework, Policy Feedback theory, Policy Diffusion theory, as well as the Narrative Policy Framework. Obviously, this list is incomplete.^2^ Rather than providing an entirely new theory, we aim at synthesizing approaches focusing on policy subsystems to carve out their contribution to the understanding of pathways to policy integration.

Regarding the politicization of policy integration in the phases of implementation and evaluation, the paper incorporates findings from research dealing with policy implementation and evaluation. We combine insights from the traditional top-down view of policy implementation (Hupe, 2011; Pressman & Wildavsky, 1984) with insights regarding bottom-up policy implementation (Agranoff, 2018; Cejudo & Michel, 2021; Gerber et al., 2009; Gollata & Newig, 2017; Maor & Howlett, 2021; Thomann & Sager, 2017). We contend that if implementation and evaluation allow the possibility for local flexibility (Dupont & Jordan, 2021; Sjöö & Callerstig, 2021; Wood, 2002), there might be bottom-up policy integration even when integration was not originally intended.

## Outputs of the process: strategies and capacities for policy integration

Policy integration is not a linear process, but multilayered and asynchronous (Candel & Biesbroek, 2016). Once initiated, it may go in different directions. In this paper, we offer explanations based on theories of the policy process, of how the actors operating in a subsystem logic open different pathways and result in different outputs. Therefore, we distinguish four possible categories of results, from non-integration (sectoral policies) to the adoption of the goals (integrated policy strategies), the means (integrative policy capacities), and the implementation practices.

1. *Sectoral policies* if policy integration initiatives are not undertaken, if they fail to reach the decision-making stage, or if they are undone during the implementation process, actors will keep working around their sector-specific policy subsystems.
2. *Integrated policy strategies* the process of policy integration may result in the adoption of integrated policy strategies. These are a set of objectives and plans for actions to overcome policy fragmentation regarding complex policy problems (Rayner & Howlett, 2009, p. 101), including a policy frame that sets a shared understanding of the problem (Candel & Biesbroek, 2016), the instruments of the policy mix being integrated (Howlett, 2019), and the responsibilities across different subsystems for achieving them (Cejudo & Michel, 2021).
3. *Integrative policy capacities* beyond enunciating strategies, the process may also result in the creation of integrative policy capacities (Candel, 2021): the skills and competencies (Wu et al., 2015), to enable policy integration policy structures and resources for keeping the policy integrated over time during the implementation. For example, policy and administrative capacities spanning across different policy subsystems, such as detailed cross-sectoral programs, coordinating bodies, or information flows to facilitate the interaction among different agencies (Cejudo & Michel, 2021; Domorenok et al., 2021, 62; Trein & Ansell, 2021). These demands compete with sectoral-specific efforts, in which actors within a subsystem would prefer to deploy sectoral policies using existing capacities. In case it is difficult to create formally integrative policy capacities, governments might end up with what we would call *loosely coupled policy capacities*. These are for example arrangements where different services agree to work together but without transforming their practices into an integrated approach (Trein, 2017), or to share information for monitoring performance but without leading to joint decision-making or pooling their resources (Cejudo & Michel, 2017). Even if there is coordination among policy subsystems, policy integration remains a secondary priority compared to their own specialization.
4. *Integrated implementation and evaluation practices* The last result refers to the practice of policy implementation and evaluation. This outcome denotes that the process of policy integration continues after formal policy decisions and also requires the integration of practices in policy implementation and evaluation between different organizations at the local or street level (Honig, 2006). Further down in the paper, we explain how this result can either occur following an integrated strategy explicitly formulated and implemented, or also bottom-up by organizations from different subsystems working across boundaries to achieve policy integration.

In the following, we turn to a discussion of pathways leading to the establishment of integrated policy strategies, integrative policy capacities, and integrated implementation practices.

## Agenda-setting and decision-making

**Fig. 1:**
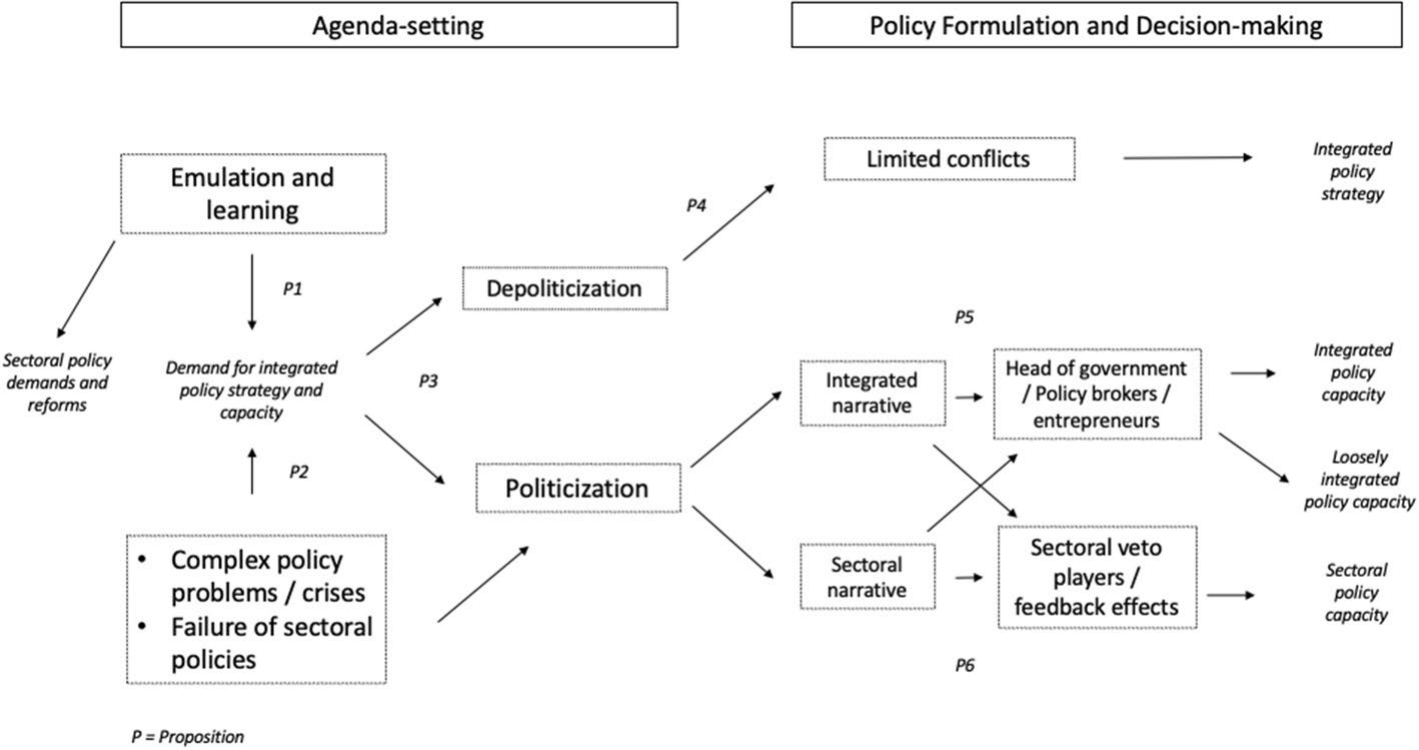
Agenda-setting and decision-making in policy integration

The process of policy integration takes place along pathways in which specific decision points determine which way the process goes. In this section, we focus on the phases of agenda-setting and decision-making in public policy. In the following, we explain the different pathways of policy integration that we propose in this paper. Figure 1 summarizes our argument.

The first element underlines how the agenda-setting process occurs outside policy subsystems, which would usually call for sectoral policies. We expect policy diffusion and external events to serve as triggers for the formulation of integrated policy proposals.

### Policy diffusion, and demands for policy integration

A pathway toward policy integration may start with demands, proposals, and ideas regarding policy changes that cut across different policy subsystems. They may be long-term projects such as climate plans (Jordan & Lenschow, 2010), but also short-term measures such as responses to crisis shocks (May et al., 2011). To understand how these ideas may activate the agenda-setting process, we hark back to the policy diffusion literature. Scholars of policy diffusion have demonstrated that decision-makers inform their agendas by looking at different jurisdictions in various ways: Policymakers emulate or learn from decisions abroad, bow to soft or hard pressure (coercion), or differentiate their agenda through competition (Maggetti & Gilardi, 2016). International organizations play an important role in policy diffusion through learning, emulation, and soft forms of coercion (Knill et al., 2001; Radaelli, 2003).

Regarding policy integration, international organizations have an important role in the formulation of policy demands as these bodies encourage nation-states to propose multi-sectoral policies aiming at dealing with pressing policy problems in a transversal way that touches upon different policy subsystems. For example, the World Health Organization pushed the “Health in all Policies” agenda (Kickbusch & Buckett, 2010), the United Nations play a key role in getting states toward the implementation of Sustainable Development Goals, (Nilsson et al., 2016), and the European Union has contributed to policy integration among its member states (Tosun et al., 2019). All these policies demand responses across several policy subsystems. Against this background, we formulate the following proposition:

#### Proposition 1

Policy diffusion, particularly from international organizations, makes integrated policy proposals likely.

### Punctuations and focusing events for policy integration

In addition to policy diffusion, punctuations and focusing events play an important role in integrated policy proposals. In the case of policy integration, such punctuations can take three forms: Firstly, they may come from a definition of policy challenges as a complex (or wicked) problem requiring a response by multiple policy sectors, such as climate change or public health (Head & Alford, 2015; Hoppe, 2011; Varone et al., 2013). Secondly, complex or transboundary crises (e.g., COVID-19) may require an integrated policy response (Ansell et al., 2010, 2020). Thirdly, policy failures (Leong & Howlett, 2021; May, 1992) may lead to the formulation of demands for integrated policies (Jochim & May, 2010).

According to different theories of the policy process, such events can trigger a process toward policy change. In the Punctuated Equilibrium Framework such events as public health crises, economic crises, or natural disasters induce politicization and policy change (Baumgartner & Jones, 2010; Baumgartner et al., 2019; Jones & Baumgartner, 2005). Such focusing events may trigger support for policy change, especially if pre-existing actor coalitions are in place (Birkland, 2006). Similarly, in the Advocacy Coalition Framework, events are an important trigger for policy change: External events or changes in adjacent policy subsystems as well as events inside a policy subsystem can activate changes in the policy agenda (Jenkins-Smith et al., 2018). Therefore, we formulate the following proposition:

#### Proposition 2

Complex policy problems and failures of sectoral policies are more likely to trigger integrated policy proposals rather than sectoral policy proposals.

### Politicization versus depoliticization regarding policy integration

According to the research on agenda-setting, policy proposals receive a lot of attention from different actors, which tend to engage in debates and conflicts regarding policy proposals (Baumgartner et al., 2018; Culpepper, 2010; Feindt et al., 2020). As discussed in the previous paragraph, punctuations can lead to the politicization of policy proposals. In addition, politicization may also depend on the type of policy instrument (Lowi, 1972). It should occur if policy proposals and projects aim at creating integrative policy capacities (Wu et al., 2015), i.e., if they assign personnel and funds for the purpose of integration to break down existing policy monopolies. If, however, the integrated policy proposal only formulates policy goals (Howlett & Cashore, 2009), for example by proposing integrated policy strategies, policy integration would remain depoliticized, since no dispute for resources would be required.

Such an instance might entail that a demand for integrated policy either receives very little public attention or that very few actors engage with it in a controversial way without many political conflicts erupting about it (de Wilde et al., 2016, p. 5). Nevertheless, depoliticization can also entail a deliberate strategy of government or other stakeholders by framing a policy demand in a way that they do not receive much political attention to avoid political contestation (Fawcett et al., 2017; Wolf & Van Dooren, 2018; Wood, 2016). One consequence of such a strategy is the adoption of integrated policy strategies, for example regarding climate change, forest protection, or digitalization, which only outline broad policy goals (Rayner & Howlett, 2009). Against this background, we formulate two additional propositions:

#### Proposition 3

If integrated policy proposals entail integrated policy strategies rather than integrative policy capacities, policy integration is likely to remain depoliticized. If they require breaking policy monopolies, the process will likely be politicized.

#### Proposition 4

If policy integration is depoliticized, governments will likely face little resistance to the adoption of integrated policy strategies, but will not create integrative policy capacities.

### Integrated and sectoral narratives for policy integration

Politicization does not automatically lead to integrated outputs. Previous research on policy integration has pointed out that framing is an important dimension of policy integration, notably whether there is an overarching frame that develops policy integration, or, whether framing remains included in an established policy sector (Candel & Biesbroek, 2016). As seen in Fig. 1, we build on this idea and acknowledge that politicization can take either a sectoral narrative or an integrated narrative. For example, an integrated policy narrative would entail that the policy setting or context, the plot, the characters, and the moral of the story (policy solution) in the policy narrative span across different established policy subsystems and connect them with each other. Contrariwise, if the elements of the policy narrative remain within an established policy subsystem, politicization can turn an integrated policy demand into a sectoral policy narrative (Shanahan et al., 2018). This competition between a sectoral and an integrated narrative regarding policy integration usually takes the shape of a conflict among boundary-spanning actors vs. subsystem veto players.

### Boundary-spanning actors versus subsystem veto players and path dependency

According to theories of the policy process, the politicization of policy issues occurs in one specific subsystem rather than by the punctuation of several policy subsystems at the same time. Put differently, communities of specialized policymakers that dominate the policy process in one policy sector (Jones et al., 1993) take over during politicization. Such “policy monopolies” give stability to policy agendas by resisting exogenously generated changes; however, they make it difficult to design policy mixes spanning across policy sectors (Howlett & del Rio, 2015). This perspective implies that once a policy issue moves onto the agenda of high politics, e.g., onto the agenda of the national government, this is likely to happen under the dominance of one specific sectoral frame (Baumgartner et al., 2018). For example, concerns related to climate change might be presented concerning energy policy because it is promising politically and would cause less resistance than a more integrated frame that combines climate policy with different policy subsystems. A recent empirical study on Swiss water policy illustrates this problem: Although policy actors engage in different issues, only a few of them focus on more than two policy issues at the same time (Brandenberger et al., 2022, pp. 48–49).

Policy integration requires breaking down several policy monopolies to combine instruments from various policy subsystems. Therefore, developing a policy proposal that creates integrative capacity requires political intervention by actors that can span across specialized policy communities. Examples of integrative policy capacities are boundary-spanning policy regimes that connect a variety of different subsystems, such as social policy or homeland security (Jochim & May, 2010; May et al., 2011). In the case of homeland security in the USA, the terrorist attacks of September 11, 2001, politicized domestic security and resulted in the establishment of policy integration capacities dealing with this important problem. There are different ways in which this process may unfold:

1. In political systems where the head of government has a high level of discretion over specialized policy communities, it is more likely that there are policy proposals that create integrative policy capacities, spanning across sectors (Alexiadou, 2015; Bäck et al., 2021; Schermann & Ennser-Jedenastik, 2014; Timmermans, 2006).
2. In a context where the head of government has less room to maneuver, other actors might play an important role in pushing policy solutions that span across different policy subsystems. According to Kingdon’s Multiple Streams Framework (Herweg et al., 2015; Kingdon, 2010; Zahariadis, 2016), policy entrepreneurs use windows of opportunities that create the political conditions to adopt the proposed solution to address the identified problem (Frisch Aviram et al., 2020). Researchers have pointed out that policy entrepreneurs play an important role in policy integration reforms (Faling et al., 2019; Trein et al., 2021a, 2021b), for instance, Green parties have played an important role in environmental policy integration in the European level (Vogeler et al., 2021).
3. This argument can be complemented by insights from the Advocacy Coalition Framework. According to this framework, the chances for integrated policies to be successfully adopted are low. In the policy process, policy subsystems in which different actor coalitions oppose one another over problems and solutions, are key to decision-making. Major policy changes are rare due to the opposition between different coalitions (Jenkins-Smith et al., 2018; Sabatier & Jenkins-Smith, 1993); however, one mechanism for overcoming stalemates between different actor coalitions is policy brokers (Ingold & Varone, 2012), which negotiate compromises between coalitions. If actors from different policy subsystems cannot agree on a policy change that would entail creating integrative policy capacities, policy brokers could contribute to generating a solution.

These theoretical elements correspond to what Candel has termed “integrative leadership” (Candel, 2021; 352). The head of government, policy entrepreneurs, and policy brokers are elements from different theories of the policy process, which authors have used and that explain why the “sectoralization” of public policies can be overcome, to create integrative policy capacities.

In contrast, if no actor is able or willing to cut across the subsystem logic, politicization will be dominated by a single policy subsystem and established policy communities, and result in the establishment of sectoral policy capacities (Fig. 1). This pathway can be explained by policy feedback theory. According to policy feedback research, elected officials, interest groups, and voters tend to support established policy structures politically, as they benefit from the status quo politically and perhaps even financially (Hacker & Pierson, 2019; Mettler & SoRelle, 2018; Pierson, 2000). If we apply this logic to policy integration, existing policy subsystems become venues for vetoes against policy integration capacities, because actors profiting from sectoral policies are afraid to lose control over policy capacities through integration. In other words, they become veto players against integration (Angelova et al., 2018; Tsebelis, 1995). From a comparative perspective, this research implies that political systems with more political constraints for the national governments should reduce the capacity for creating integrative policy capacities, although the empirical results in that regard are inconclusive (Trein & Ansell, 2021).

The discussed theories regarding policy narratives, policy entrepreneurs, brokers, and heads of governments as well as feedback effects and veto players imply additional theoretical considerations. Despite the presence of an integrated policy narrative, one important sectoral veto player might block the creation of integrative policy capacities. On the contrary, even if there is a sectoral policy frame due to persisting political conflicts about integration, boundary-spanning actors might use their political leadership to establish integration. If the results of these conflicts are inconclusive or become negotiations achieving compromises, the outcome may be loosely integrated policy capacity. This result entails that some efforts are made to improve coordination, sharing information, and orienting actions toward commons goals, but there are no new specific instruments, shared resources, or new sources of authority. Notably, cross-sectoral forums could be a place for such exchanges (Fischer & Maag, 2019).

These theoretical discussions imply two further propositions regarding pathways of policy integration in the policy process:

#### Proposition 5

If policy entrepreneurs, policy brokers, or heads of government make policy integration a priority, the likelihood of integrative policy capacities increases. They might even turn a sectoral narrative into integrated or loosely coupled policy capacities.

#### Proposition 6

If veto players and feedback effects from policy subsystems dominate in the policy process, the likelihood of integrative policy capacities decreases. They may even turn an integrated policy narrative into sectoral policy capacities.

## Implementation and evaluation

**Fig. 2:**
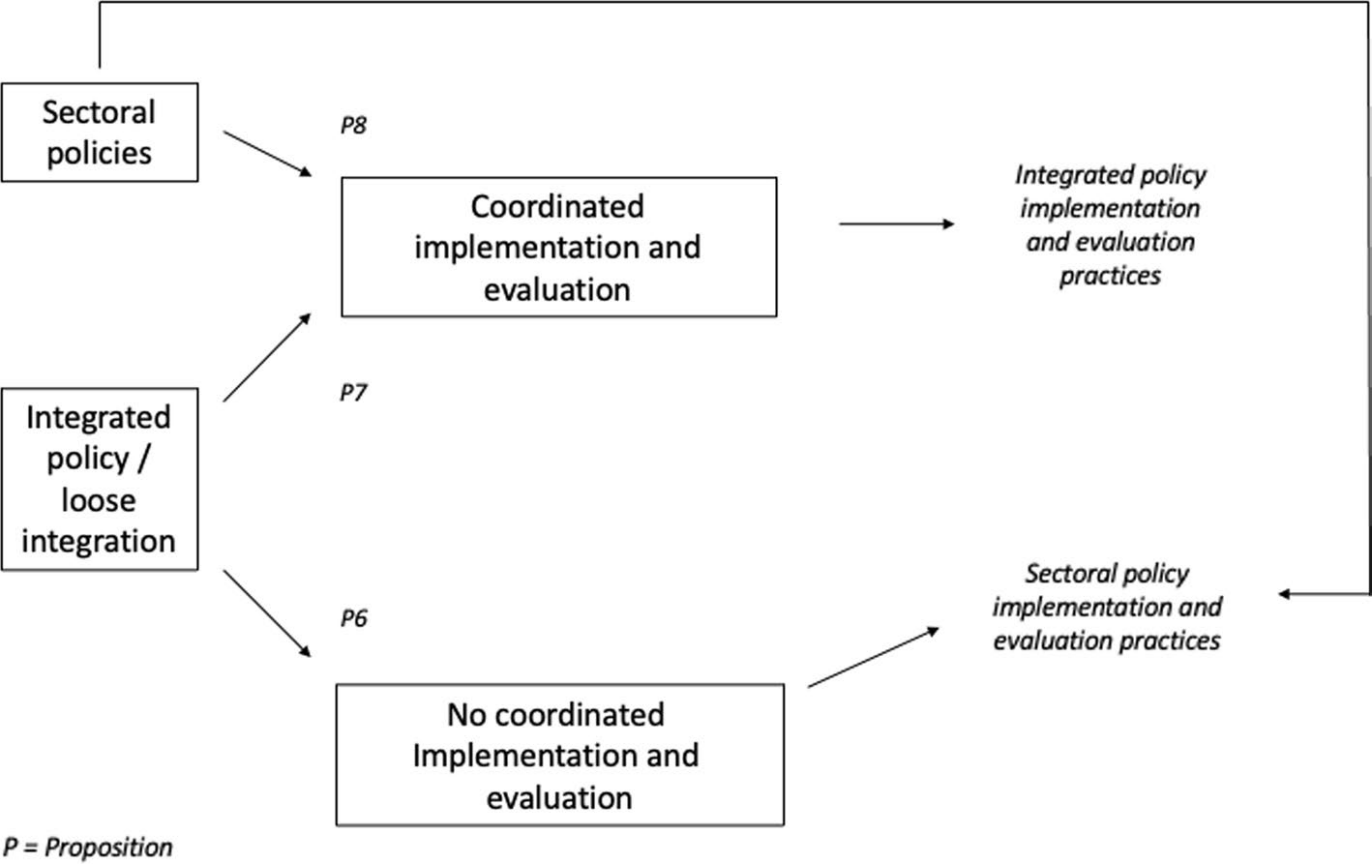
Implementation and evaluation in policy integration

Once a decision has been made about whether an integrated policy or sectoral policies will be pursued, the implementation and evaluation phase will shape the outcomes of the process. As seen in Fig. 2, we anticipate three pathways with two possible outcomes. If there was no decision for an integrated policy strategy or integrative policy capacities, the default endpoint would be sectoral policymaking. Similar to decision-making, implementation, and evaluation of public policies tend to be sector-specific: within policy, subsystems where organizations address policy problems with the instruments, resources, methodologies, and routines they have at hand (Fischer, 2019; Hill & Varone, 2021; May, 2015; Pawson & Tilley, 1997).

Thus, practices to implement integrated policy strategies and integrative capacities are likely to face the classical challenges of policy implementation: a *joint-action problem* and a high number of veto possibilities if many actors are involved in policy implementation (Pressman & Wildavsky, 1984). This veto position of implementing agents makes more “incongruent implementation” likely, especially (a) if authority is decentralized (that is, traditional command-and-control mechanisms are not in operation because implementing actors have independent authority), and (b) if policy rules and goals are ambiguous (Hupe, 2011; Sabatier & Mazmanian, 1980). Since policy integration often entails the inclusion of many different program-level policy goals, it becomes difficult for implementers to identify their contributions to the overarching goals of the integrated strategy and the likelihood of policy failure increases (Vince, 2015). Therefore, conflicts and previously established patterns of interaction between implementing agencies might undermine the implementation of integrated policies (Mavrot & Hadorn, 2021; Peters, 2015). These insights imply for policy integration that integrated implementation or evaluation practices are unlikely because implementing organizations follow their established sectoral practices and seek to preserve their autonomy.

### Proposition 7

The prevalence of joint-action problems by implementing agencies makes sectoral rather than integrated implementation and evaluation more likely.

Yet, it is not only the passive acquiescence of several subsystems that leads to coordinated implementation and evaluation. The classical solution to the implementation problem proposed by Pressman and Wildavsky was to eliminate program complexity or reduce the number of veto players (Pressman & Wildavsky, 1984). Direct supervision (May, 2015) or performance indicators and targets (Hood, 2006) could be attempted to steer the congruent implementation of policy. In the case of policy integration, there is a limit to this approach, given that, by definition, different policy subsystems will be involved. An integrated policy involves a policy mix with different instruments being deployed by agencies from different policy sectors. The implementation of such a policy mix involves more than the mere simultaneous implementation of parallel policies, but the interaction among those policies (Cejudo & Michel, 2021; Gerber et al., 2009; Maor & Howlett, 2021). These attributes have important implications for the study of its implementation. Different contributions to the literature have suggested that implementing public policy programs that span across various sectors is difficult due to potential conflicts or simply a lack of congruence between involved organizations (Candel, 2017; Gerber et al., 2009). The challenge with implementing integrated policies is that they involve more than one agency and sector, which creates additional horizontal coordination challenges (Egeberg & Trondal, 2016). Thus, implementation is also a political process. Implementing integrated public policies not only requires designing sectoral coherence at the level of the policy program but also preventing conflict and facilitating congruence between different implementing agencies that respond to the logic of their own policy subsystems.

### Proposition 8

The availability of integrative capacity to steer the process of implementation makes it more likely that coordinated implementation and evaluation practices will occur.

Finally, integrated implementation and evaluation practices might emerge in a bottom-up fashion, even when the starting point is sectoral policymaking. For example, in decentralized political systems, lower levels of government often have the discretion to shape policy implementation according to their own functional and political needs (Gerber et al., 2009; Gollata & Newig, 2017; Thomann & Sager, 2017), which may even increase the effectiveness of public policies (Knill et al., 2021). This logic implies that local politicians, street-level bureaucrats, or middle managers might not only veto but alter the logic of implemented policies.

In contrast to the top-down approach to the study of implementation and evaluation, the bottom-up perspective sees the process of implementation not as the inevitable deviation of a perfect plan, but as an ongoing process of policymaking: Organizations, managers, and street-level bureaucrats are not passive receptors of policy directives, but actors that shape the policy process (Lipsky, 2010) as the implementation of a policy requires a group of people and organizations capable of acting following what is envisaged. From this bottom-up view, there is always the possibility that the actors have sufficient margins of action to be able to orient their actions to the purpose of the policy (Hupe et al., 2015), recreate the causal theory, and modify the objectives of a given policy. This implies that if implementing organizations recognize the importance of policy integration, the synergies derived from cooperation, or the gains in efficiency from collaborative arrangements, they may create integration from below. They overcome the subsystems’ restrictions and move toward greater integration through integrated practices of policy integration (Lidén & Nyhlén, 2021), even if formal policy integration is absent. Furthermore, evaluation tools might contribute to such a style of bottom-up policy integration. For example, evaluation tools such as “Environmental Impact Assessments” or “Sustainability Impact Assessments” examine the impact of any sectoral policy regarding its consequences for environmental protection and sustainable development. Such policy evaluation tools at the disposal of decision-makers are potential drivers of policy integration as well (Dupont & Jordan, 2021; Wood, 2002), by assessing the coherence among policy interventions and identifying where expected synergies occurred or not (Sjöö & Callerstig, 2021). Therefore, we formulate the following proposition, which highlights the nonlinearity of the process of integration, and the multiple ways in which the pathways may result, or not, in integrated policy practices:

### Proposition 9

Flexibility and incentives for public managers, street-level bureaucrats, and local governments may increase the likelihood of integrated implementation and evaluation practices, even in the absence of national integrated policy strategies and capacities.

## Conclusions

In this article, we contribute to the literature by using theories of the policy process (Weible & Sabatier, 2018) to understand the role of actors within policy subsystems and the logic of politicization in policy integration. We explain how the pull effect of established policy subsystems impacts politically on policy subsystem integration in policy change. Building on previous research that has underlined the processual nature of policy integration (Candel & Biesbroek, 2016; Cejudo & Michel, 2017), we have discussed how policy integration is placed in the policy process, starting from the inclusion of integrated policy programs on the decision-makers' agenda, to the decision-making process regarding integrated policy strategies and integrative policy capacities, and the implementation and evaluation practices concerning policy integration.

The contribution of our paper is theoretical. By identifying propositions about how the process of integration unfolds, we have provided scholars with a set of propositions that could guide empirical inquiries about the dynamics of the policy process through which integration takes place. Such empirical analysis could take politicization more seriously in the process of policy integration, which is different from previous analyses that have emphasized design aspects of policy integration (Howlett & Mukherjee, 2018; van Geet et al., 2021). We anticipate that such empirical research will refine the propositions offered here, pose new questions to those theories, and identify new, complementary pathways related to the politicization of policy integration. Given the nonlinearity of the policy integration process, it is not expected that specific propositions could automatically be linked to one of the pathways (or its outcomes), but empirical, comparative research may shed light on the mechanisms at work.

Which are then the next steps for the policy integration research agenda? Firstly, scholars may put the propositions developed in this paper to an empirical test. Secondly, by using such empirical insights scholars could link the Institutional Analysis and Development Framework (Ostrom, 2011), the Ecology of Games Framework (Lubell, 2013), or other theories to the insights in this paper. Finally, as new theories of the policy process are developed (Cairney & Weible, 2017; Petridou, 2014) and applied to new contexts and conditions (Weible et al., 2020), new lessons will be drawn to improve our explanations about how governments carry out policy integration in different sectors and countries. The research agenda should also pay more detailed attention to the mechanisms at work (van der Heijden et al., 2021) and the feedback effects of politicization.

## Footnotes

1 For an exception cf. (Trein, Maggetti, et al., 2021a, 2021b).

2 We exclude approaches of policy analysis that explicitly address the complexity of policymaking, such as the Institutional Analysis and Development Framework (Ostrom, 2011) and the Ecology of Games Framework (Lubell, 2013). These approaches focus on the complexity of institutional structures and do not pursue an explicit subsystem approach. Our contribution complements these frameworks.
